# Evaluation of a smartwatch-based intervention providing feedback of daily activity within a research-naive stroke ward: a pilot randomised controlled trial

**DOI:** 10.1186/s40814-018-0345-x

**Published:** 2018-10-06

**Authors:** Sophie Lawrie, Yun Dong, Dax Steins, Zhidao Xia, Patrick Esser, Shanbin Sun, Fei Li, James D Amor, Christopher James, Hooshang Izadi, Yi Cao, Derick Wade, Nancy Mayo, Helen Dawes, Smart Watch Activity Feedback Trial Committee (SWAFT), Lingzhi Wu, Peifang Li, Ying Wang, Chong Chen, Peiyang Sun, Jinji Wang, Feifei Wang, Panfu Hao, Weiwei Wu, Yubao Gao, Xiaoli Sun, Haiyang Wu, Yujie Yang, Yuanfeng Peng, Jingjing Xue, Xiaoli Guo, Xuesong Xie, Na Zuo, Xinkui Gao

**Affiliations:** 1 Centre for Movement, Occupational and Rehabilitation Sciences (MOReS), Department of Sport, Health Sciences and Social Work, Oxford Brookes University, Headington Campus, Headington Road, Gipsy Lane, Oxford, OX3 0BP UK; 2 Rehabilitation Centre, the Second Affiliated Hospital of Anhui University of Traditional Chinese Medicine, Hefei, Anhui Province China; 3 School of Engineering, University of Warwick, Coventry, CV4 7AL UK; 4 School of Physical and Occupational Therapy, McGill University, 3654 Prom Sir-William-Osler, Montréal, Québec H3G 1Y5 Canada

**Keywords:** Stroke, Physical activity, Activity feedback, Feasibility, Research naive, Rehabilitation

## Abstract

**Background:**

The majority of stroke patients are inactive outside formal therapy sessions. Tailored activity feedback via a smartwatch has the potential to increase inpatient activity. The aim of the study was to identify the challenges and support needed by ward staff and researchers and to examine the feasibility of conducting a randomised controlled trial (RCT) using smartwatch activity monitors in research-naive rehabilitation wards. Objectives (Phase 1 and 2) were to report any challenges and support needed and determine the recruitment and retention rate, completion of outcome measures, smartwatch adherence rate, (Phase 2 only) readiness to randomise, adherence to protocol (intervention fidelity) and potential for effect.

**Methods:**

First admission, stroke patients (onset < 4 months) aged 40–75, able to walk 10 m prior to stroke and follow a two-stage command with sufficient cognition and vision (clinically judged) were recruited within the Second Affiliated Hospital of Anhui University of Traditional Chinese Medicine. Phase 1: a non-randomised observation phase (to allow practice of protocol)—patients received no activity feedback. Phase 2: a parallel single-blind pilot RCT. Patients were randomised into one of two groups: to receive daily activity feedback over a 9-h period or to receive no activity feedback. EQ-5D-5L, WHODAS and RMI were conducted at baseline, discharge and 3 months post-discharge. Descriptive statistics were performed on recruitment, retention, completion and activity counts as well as adherence to protocol.

**Results:**

Out of 470 ward admissions, 11% were recruited across the two phases, over a 30-week period. Retention rate at 3 months post-discharge was 48%. Twenty-two percent of patients dropped out post-baseline assessment, 78% completed baseline and discharge admissions, from which 62% were assessed 3 months post-discharge. Smartwatch data were received from all patients. Patients were correctly randomised into each RCT group. RCT adherence rate to wearing the smartwatch was 80%. Baseline activity was exceeded for 65% of days in the feedback group compared to 55% of days in the no feedback group.

**Conclusions:**

Delivery of a smartwatch RCT is feasible in a research-naive rehabilitation ward. However, frequent support and guidance of research-naive staff are required to ensure completeness of clinical assessment data and protocol adherence.

**Trials registration:**

ClinicalTrials.gov Identifier, NCT02587585–30th September 2015

**Electronic supplementary material:**

The online version of this article (10.1186/s40814-018-0345-x) contains supplementary material, which is available to authorized users.

## Background

Exercise has an important role in the recovery of stroke, increasing cognition, arm function, balance and gait, in addition to reducing the risk of subsequent cardiovascular events [1, 2]. Despite the importance of general physical activity in recovery, the majority of stroke survivors receiving rehabilitation in hospital are inactive outside formal therapy sessions [3, 4]. In order to encourage long-term exercise adherence, it is recommended that physical activity goals are customised to the individual tolerance of the stroke patient [2].

Modern electronic activity monitors are able to provide a wide range of behavioural monitoring tools and are therefore emerging as a possible method to provide customised activity goals and feedback to promote exercise [5]. Coinciding with the technological developments in activity monitoring, there is evidence to suggest that activity feedback of exercise may increase motivation to exercise. The provision of activity feedback has been found to be more effective in increasing physical activity levels than providing activity goals alone, in healthy controls [6–8] and in older adults undergoing rehabilitation [9]. Interventions providing feedback and monitoring of activity have shown positive outcomes in relation to exercise adherence amongst older individuals [10]. However, personalised activity feedback has also found to have no effect on actual or intended activity levels amongst controls [11]. Despite studies suggesting a positive effect, more evidence is needed before such activity feedback interventions can be recommended to be used in treatment.

The literature has shown that remote monitoring of physical activity is feasible after stroke [12]; however, the impact of activity feedback on exercise levels within this population is less clear. A systematic review of studies investigating augmented feedback on motor activities after stroke concluded that findings were inconsistent due to the combination of multiple aspects and types of augmented feedback used [13]. One study found that feedback of physical activity provided three times a week had no significant effect on the daily walking time of stroke inpatients [14]. Little research to date has investigated the use of periodic feedback of daily activity amongst stroke patients undergoing rehabilitation. It is of interest to see whether increasing the frequency of activity feedback will elicit greater physical activity levels. The provision of daily activity feedback (via a smartwatch), relative to activity at fixed time points through-out the previous day, may have the potential to motivate stroke rehabilitation patients to be more active.

Conducting clinical trials within research-naive settings are commonly accompanied with ethical, cultural and organisational challenges [15]. The present study will evaluate the feasibility of conducting the smartwatch intervention mentioned above within a research-naive stroke rehabilitation centre in Hefei, China (whereby no rehabilitation research has previously been conducted).

The aim of this feasibility study was to identify the challenges and support needed by ward staff and researchers and to examine the feasibility of conducting an RCT using smartwatch activity monitors in research-naive rehabilitation wards. The objectives were to report any challenges and support needed and determine the recruitment and retention rate, completion of outcome measures, adherence to wearing the smartwatch, readiness to randomise, adherence to protocol (intervention fidelity) and potential for effect. 

## Method

### Research design

**Fig. 1:**
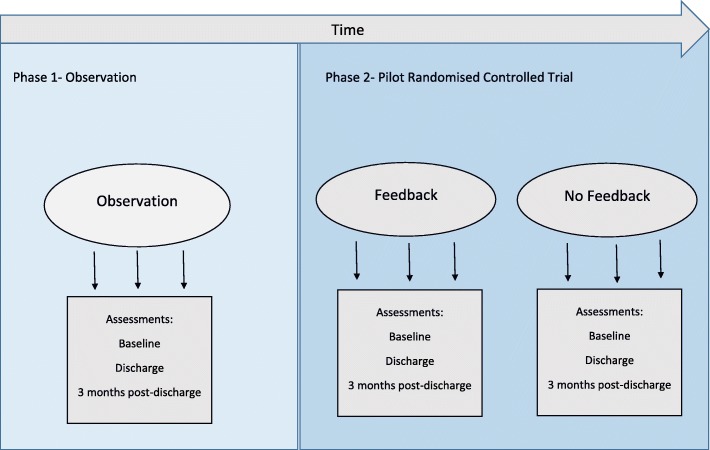
Schematic representation of the research design showing two phases (observation and pilot Randomised controlled trial (pilot RCT)) and three groups- observation, feedback and no feedback

The research design included two experimental phases, an observational phase and a pilot RCT (see Fig. 1). Criteria for participant recruitment and selection were identical between the two phases (observation and pilot RCT). Group allocation into either phase was determined by the time from study onset. In keeping with the allocated period of funding and to allow the maximum time for the pilot RCT phase, patients were recruited into the observation phase 1 month from trial onset and the pilot RCT from 2 to 6 months from trial onset.

#### Phase 1: Observation phase

The observation phase was a non-randomised (non-blinded) experimental phase. Research assessors and ward staff were not blinded as all patients received no activity feedback. The protocol for the observation phase was identical to that of the pilot RCT phase, except that there was only one group (no patients received feedback). Patients recruited into the observation phase were provided with a smartwatch that recorded their activity but provided no feedback about the physical activity levels during a 3-week intervention period.

#### Phase 2: Pilot randomised controlled trial phase

Phase two was a single-blind, pilot randomised controlled trial; with the research assessor blinded to the group allocation (it was not possible to blind the ward staff and patients). Group allocation was performed by an independent researcher using a 1:1 allocation ratio. A computer-generated random sequence was generated in Microsoft Excel to allocate groups and generate numbers of which was then used to assign participants. The patients recruited into the pilot RCT phase were randomly assigned using concealed allocation in envelopes held centrally, into one of two groups:

- To receive feedback on the amount of physical activity (movement) undertaken, two hourly for the first 8 h and then for the last 1 to 2 hours (dependent on the battery life) or
- To receive no feedback about the physical activity levels during a 3-week intervention period.

Once recruited, the research office (unblinded) was informed by the recruiting doctor and the research office then registered the patient, opened the next envelope and notified the ward (unblinded) about the patient’s group by phone. The researcher assessors performing all pre- and post-assessments remain blinded to group assignment throughout the duration of the study.

### Aims and objectives

The study consisted of two phases, an initial observation phase (phase 1) and a pilot randomised controlled trial (phase 2). Each phase shared the same overall aim: to identify the challenges and support needed by ward staff and researchers and examine the feasibility of conducting an RCT trial using smartwatch activity monitors in stroke survivors in research-naive rehabilitation wards.

The objectives per phase were as follows:

- Phases 1 and 2: to report any challenges and support needed and determine the recruitment and retention rate, completion of outcome measures and adherence rate to wearing the smartwatch
- Phase 2 only: determine the readiness to randomise, adherence to protocol (intervention fidelity) and potential for effect.

Patients that received activity feedback (pilot RCT- feedback group) were expected to exhibit increased activity levels than patients who did not receive activity feedback (observation group and pilot RCT no feedback group). It would also be expected that increased activity will result in improved mobility, and possibly cognition, arm function, independence in daily activities and health-related quality of life within the feedback group. However, no significant differences between groups in activity or clinical assessment outcomes are expected in this trial as this is a feasibility study and thus underpowered.

### Research process

The study involved multiple clinical research groups within the Second Affiliated Hospital of Anhui University of Traditional Chinese Medicine (ward staff (doctors and nurses), research assessors, the Smart Watch Activity Feedback Trial Committee (SWAFT) and research officers) and those of the Oxford Brookes Centre for Movement, Occupational and Rehabilitation Sciences’ (MOReS) and Warwick Biomedical Engineering group. The SWAFT team members are listed in the title page and met weekly to resolve any local practical problems involving the study. The ward staff conducted the smartwatch intervention and screening of participants, and the research assessors conducted the clinical assessments and oversaw the maintenance of research databases and transfer of research data.

Before this study started, the Oxford (UK) research team visited the host hospital in Hefei twice to develop the research ideas, design and governance with the local Chinese clinical and research group. Then, specific protocols (standard operating procedures) were written, ethical applications sought in local institutional settings, and a 2-week formal training in the execution and governance of the protocols, procedures for data collection and recruitment was given to the ward staff and the research assessors. The full protocol can be accessed online [16].

Specific roles within the study were as follows:

- Ward staff:
  - Identify and record new ward admissions
  - Assess and record eligibility
  - Recruit patients onto the study (recruiting doctor)
  - Inform the research office of the above
  - Obtain informed consent (day 0)
  - Re-checking eligibility on patients who were failing the initial clinical eligibility test (day 3)
  - Initiating trial—providing smartwatch and explaining its function (day 1)
  - Preparation, distribution and collection of smartwatches and download of data
  - Checking patient after first 8 h of intervention—one working day (day 1)
  - Daily review of patients receiving intervention for adverse events—both groups
  - Inform research office of any adverse events
  - Record any patient feedback
- Unblinded members of the research office:
  - Register new patients onto trial database
  - Notify the ward about a patients group allocation
  - Fortnightly teleconference calls and email data transfer with the Oxford research team
  - Receive and file consent forms
  - Record ineligible patients
  - Collation of data
- Research assessors:
  - Conduct clinical assessments and record outcome data (in ward and via telephone at 3 months)
  - Record adverse events
- Oxford and Warwick research teams:
  - Receiving and securing data
  - Data analysis
  - Technical support regarding gait sensor and smartwatch

To monitor and provide support to increase fidelity with the research protocol, a further visit after 3 months was undertaken and then regular video-conferencing calls were made every 2 weeks, and data were sent via protected email every 2 weeks to Oxford for storage and analysis. When required, the Warwick group provided technical support for the smartwatch by email and video-conferencing calls which helped to eliminate some early issues with the smartwatch application and the way it was being used. In these ways, as much support and help as possible was delivered and the quality and completeness of data were monitored.

### Participant selection and recruitment

Patients admitted across four rehabilitation wards in the Second Affiliated Hospital of Anhui University of Traditional Chinese Medicine, Hefei, Anhui Province, China were recruited by a recruiting doctor that is based on the ward. Recruitment numbers were subject to the number of admissions to the wards within the allocated 6-month completion period of the study. All patients included in this paper were recruited onto the study before 30th April 2016. Criteria for participant recruitment and selection are identical between the two phases (observation and pilot RCT). Recruitment into the pilot RCT occurred after the observation phase had stopped recruiting patients.

Patients were eligible for screening if admitted after a recent stroke (less than 4 months), and the following questions were answered positively:

1. Can the patient follow a two-stage command, judged clinically?
2. Was the patient walking without help from another person before this stroke?

Recruitment was dependant on whether the patient satisfied demographic and performance-based inclusion criteria.

Demographic inclusion criteria are as follows:

- are aged 40–75 years
- are less than 4 months after stroke onset
- are having their first admission for rehabilitation
- were able to walk at least 10 m prior to stroke, without help of another person; use of equipment allowed

Performance-based inclusion criteria are as follows:

e) have sufficient cognition to participate in study and testing procedures (clinically judged, because there are no valid short cognitive measures to assess ability to consent and participate)
f) can follow a two-stage command (e.g. pick up an object, put it on the table)
g) has sufficient visual function to see watch feedback (clinically judged)
h) have the capacity to consent and do give consent to the study

If a subject matched all other inclusion criteria but was unable to follow a two-stage command, they would be reassessed at a 3-day follow-up and included if the criteria were then met during this time. Only first-time admissions into the stroke ward were accepted, but the stroke could be a second or subsequent stroke provided the patient met the inclusion criteria. A member of the ward staff responsible for recruiting explained the consent form to eligible patients allowing ample time for full comprehension and to answer any questions. All patients were given 24 h to decide if they wanted to participate in the study. Fully informed written consent was then obtained from willing participants in the form of a signature.

### Intervention

#### Clinical assessments

Upon admission to rehabilitation, the standard demographic data collected on each patient as part of routine care included age, gender, weight, height and grip strength in the unaffected arm (left or right). Routine hospital data were used to record total the number of patients admitted after stroke and the dates of stroke onset. All patients received a local routine that includes drug treatments, acupuncture (with additional Moxibustion, cupping and Tui Na (Massotherapy)) and rehabilitation. The drug and acupuncture treatment are performed in the morning between 9:00 and 11:00 am prior to rehabilitation performed in the afternoon and included the following:

- Drug treatment: routine neuron stimulation and nutritional therapy (Cerebrolysin, Vinpocetine or Ganglioside) and herb medicine for ‘promoting blood circulation by removing blood stasis’, and other associated symptoms or complications were administered to all cases included in the study.
- Acupuncture treatment: acupuncture treatments were performed according to patients’ condition based on traditional medical diagnosis, with additional Moxibustion, cupping or Tui Na therapy if needed.
- Rehabilitation: Following functional evaluation of each patient, a rehabilitation treatment plan was established accordingly. The treatment will include physiotherapy, occupational therapy and physical factor therapy—including low frequency, intermediate frequency, magnetic heat and transcranial magnetic.

Patients with stroke within the Second Affiliated Hospital of Anhui University of Traditional Chinese Medicine are typically discharged from in-patient rehabilitation between 2 to 3 weeks, and thus the length of intervention differed between subjects. All patients entered into the research study were assessed at baseline (0 weeks) and at discharge from the ward (≤ 3 weeks) by a research assessor, who did not know their group allocation, on:

- Cognition, using the Montreal Cognitive Assessment (MoCA) [17]—Mandarin version [18, 19]
- Activities of daily living, using the Barthel ADL index [20] and the World Health Organization Disability Assessment Scale (WHODAS) 12 item version [21]
- Fatigue, using the Fatigue Severity Scale [22, 23] and a visual analogue scale with anchor points of ‘no experience of fatigue’ (= 0 mm) and ‘worst fatigue imaginable’ (= 100 mm)
- Quality of life, using the EuroQol 5D-5L (EQ-5D-5L) and its visual analogue scale with anchor points of ‘best state of health you can imagine’ (100) and ‘worst state of health you can imagine’ (0) [24, 25]
- Measures of mobility; Rivermead Mobility Index (RMI) [26] and a 10-m walk test (10MWT) time [27]
- Grip strength [28]; that provides a quantitative and objective measure of isometric muscular strength of the hand and forearm
- Spatio-temporal gait features at self-selected walking speed measured during the 10MWT using an inertial sensor on the lower trunk [29]. Participants were instructed to walk twice along a 10-m walkway at their normal pace with a walking aid or support of a researcher. Walking speed, cadence, step length and symmetry of spatio-temporal measures were calculated.

Test batteries and associated instructions were translated into Mandarin. The final assessments were at 3 months post-discharge by telephone (one telephone call was made) and included the WHODAS, RMI and EQ-5D-5L and its visual analogue scale only. The visual analogue scale was completed by verbal instruction via telephone. The following was translated into Mandarin: ‘We would like to know how good or bad your health is TODAY. On a scale numbered from 0 to 100, where 100 means the best health you can imagine and 0 means the worst health you can imagine, what would you rate your health today?’

### Smartwatch activity recording

The smartwatch was instructed to be worn throughout the day (08:00–17:00) in the rehabilitation ward from Monday to Friday for up to 15 days (21 days elapsed in total) or until discharge if sooner. A member of the ward staff provided each patient with a ZGPAX S8 Android™ [30] smartwatch each morning, to wear on the unaffected side. The watch divides elapsed time into four periods of 2 h and one period of 1 h (to match the duration of battery life). The first day served as a baseline measure for each time period, where none of the patients in any phase received feedback on their levels of activity. Ward staff were instructed to explain to patients in the feedback group that over a 2-h period, the watch would indicate progress towards the activity goal for those 2 h. For patients in the observation phase and the no feedback (control) group, ward staff were required to explain that activity scores were recorded on the watch but otherwise provided no feedback or motivating comments. Each day after baseline, the smartwatch would automatically set the goal based on a 5% increase in the total activity recorded in the comparable 2-h epoch the preceding day (Friday was used for the Monday goals). In the no feedback group, the activity level is simply recorded and no further processing or action occurs. The research doctors were instructed to ask the patients about any problems with the watch and to look out for any observable adverse effects such as a skin rash. Each evening at about 17:00, a member of the ward staff removed the watch, downloaded the data into a computer through a USB connection and recharged the watch battery overnight using the same USB connector. Encrypted smartwatch and assessment data were sent from Anhui research office by protected email to the Oxford research team upon completion.

The ZGPAX S8 Android™ smartwatch runs a custom-built activity monitoring and user feedback application established from previous work investigating physical activity in the elderly [31, 32]. The smartwatch specifications are shown in Additional file 1. Data from the ZGPAX S8 tri-axial accelerometer is processed to extract a measure of overall body movement, the activity score, alongside an estimate of whether the watch was or was not worn [33]. Activity score has been shown to correlate highly (*p* < .001) with energy expenditure obtained from whole room calorimetry [34]. The intention of the watch is to compare relative levels of activity from day to day rather than a specific measurement of actual activity. A higher activity score can be achieved by either very intense short bursts or very low intensity yet continuous movement. Activity was not summated into standardised thresholds of passive, moderate or vigorous exercise as this has been shown to be inappropriate for individuals with stroke [35].

**Fig. 2:**
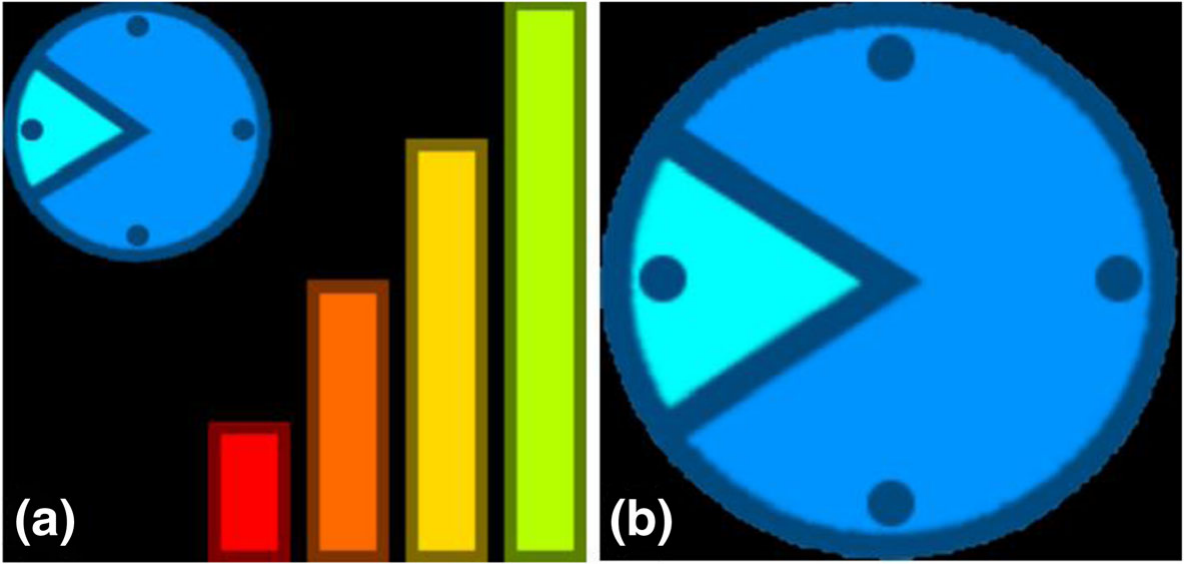
Activity feedback as displayed on the smartwatch screen for the feedback group (**a**), which included both the feedback bars and clock face, and the no feedback (control) group (**b**), which included the clock face only

**Fig. 3:**
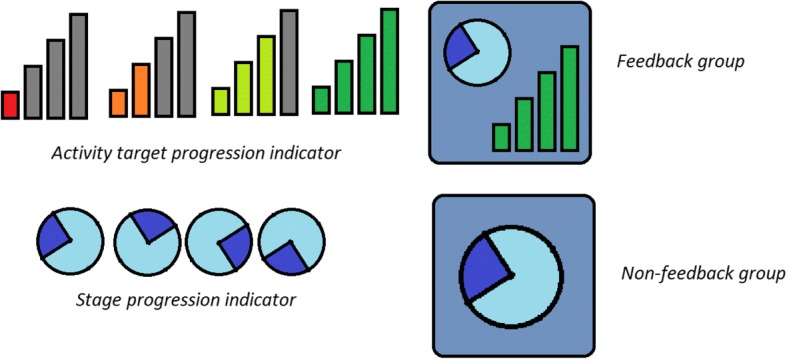
Visual representation of the feedback provided on the smartwatch

As there is little research using feedback in this manner, no previously validated feedback icons were available. The feedback icons used in the smartwatch software were developed in collaboration between Warwick and Oxford Brookes with the aim of providing feedback on both activity levels and the current daily time period. Feedback was displayed visually in the form of four progressive bars (see Fig. 2). The bars act as an activity tracker to indicate the amount of activity completed relative to their goal. The bars differ in size and colour from red to green, depending on how close the patient is to completing their activity goal. Bar 1 is highlighted red as default, signifying little or no activity. Bars 2, 3 and 4 correspond to completion of 1/3 (orange), 2/3 (yellow) and 3/3 (green) of the activity goal respectively. The clock icon shows the patient which of the five time periods (between 08:00 and 17:00) they are in and therefore roughly the current time. Visual presentation of the smartwatch within the control (no feedback) group consisted of the clock icon only. Visual comparisons of the icons displayed by the smartwatch within the feedback and control group are shown in Fig. 3.

### Data analysis

Data analysis involved descriptive statistics performed on recruitment, retention and completion, activity counts as well as adherence to protocol. All data analysis was conducted by the Oxford and Warwick research teams. The current study is an external pilot, and therefore, the data collected will not form part of a subsequent main trial. The data collected in both phases included the following:

- Challenges and support needed by ward staff and researchers (office and assessors)
- Recruitment rate (percentage of patients who were recruited out of total patients admitted for rehabilitation after stroke)
- Retention rate (percentage of patients that completed assessments at baseline, discharge and follow-up out of the total number of patients recruited)
- Duration in days between stroke event and admission and from admission to trial registration
- Duration in days from the start of the intervention to discharge and admission to discharge
- Range of data completion per outcome
- Process for acquiring, recording, transferring and quality control of original trial data
- Adherence rate to wearing the smartwatch (days the smartwatch was worn/total days of data received)
- Adherence to protocol by research assessors and ward staff (standard operating procedures)
- Readiness to randomise (number of patients randomised and recorded as per the protocol)
- Potential for effect (number of days that patients exceeded their baseline activity level per group)
- Any feedback from patients, ward staff and research team
- Self-reported and observed adverse events (from patients and ward staff).

A patient’s activity was considered to have exceeded baseline activity if the total daily activity score recorded was greater (*n* > baseline) than the activity score recorded at baseline (day 1 of the intervention).

### Ethics

This study was sponsored by the Second Affiliated Hospital of Anhui University of Traditional Chinese Medicine. The study was approved by the Chinese Ethics Committee of Registering Clinical Trials (ChiECRCT-20150034), West China Hospital, Sichuan University, 37 Guoxuexiang, Chengdu, Sichuan, China. The study was registered at the Chinese Clinical Trial Registry on 08 August 2015, number ChiCTR-IOR-15007179 and on clinical trials.gov (NCT02587585; 30 September 2015).

All research was in compliance with the Helsinki Declarations and the Research Governance Framework for Health and Social Care. Informed written consent was obtained by a recruiting doctor based on the wards, whereby sufficient mental capacity (through clinical judgement) and understanding of anonymity and the inclusion of their data was ensured by verbal confirmation.

## Results

### Research challenges and support needed by ward staff and researchers

The major challenges when conducting this research included the following:

- Maintaining consistent recording and transfer of data
- Maintaining communication between the research and ward teams to avoid loss of participants (to notify when patients are to be discharged and thus require assessment)
- Ensuring clear understanding of specific roles within the research office, assessors and ward staff
- Language barriers between the English and Chinese teams that made it difficult to explain fine details of data collection/transfer.

Specific support needed by the research office, research assessors and ward staff included the following:

- More clarity in individual roles
- More emphasis/understanding on the importance of complete data both from research assessors and patients
- A bilingual individual overseeing the transfer of data proficient in Mandarin and English to ensure full comprehension
- Email reminders to send over the research data files
- Regular surveillance of the data collected to ensure consistent data collection.

### Recruitment and retention

Between September 22, 2015, and April 19, 2016, the hospital admitted 470 patients for rehabilitation after stroke and 51 (11%) were recruited into the two phases. One patient was registered accidentally (they did not meet the inclusion criteria of sufficient cognition), leaving 50 valid patients. Patients in the observation phase started the intervention between October 15, 2015, and November 11, 2015. Patients in pilot RCT started the intervention from November 11, 2015 until April 22, 2015. Demographics are shown in Table 1.

**Table 1:** Patient demographics per group (mean (SD))

	Observation (*n* = 20)	Pilot RCT	
		Feedback (*n* = 14)	No feedback (*n* = 16)
Age (years)	61 (9)	53 (12)	62 (12)
Gender (n)			
Male	14	10	13
Female	6	4	3
Affected side (*n*)			
Right	7	7	5
Left	13	7	11
Duration from Stroke event to admission (days)	74 (50)	42 (25)	50 (29)
Duration from admission to trial registration (days)	8 (7)	7 (14)	5 (10)
Duration from the start of the intervention to ward discharge (days)	Unknown*	18 (7)	17 (8)
Total days from admission to ward discharge (days)	Unknown*	26 (17)	22 (11)

*Due to missing discharge dates

**Fig. 4:**
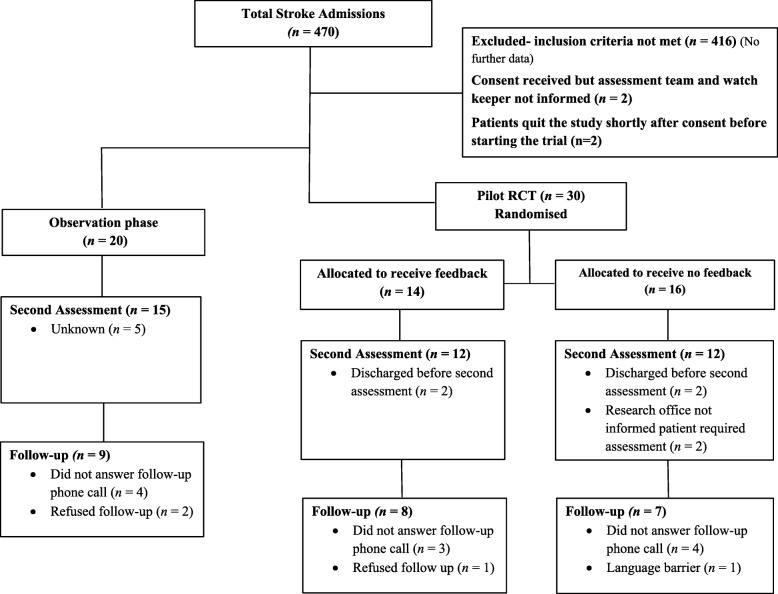
Participant recruitment within the observation and the pilot randomised controlled trial (RCT) phase

The percentage of participants retained at discharge was 78% (39 out of 50 patients) and at 3 months post-discharge was 48% (24 out of a total 50 patients).The last of the 3-month follow-up telephone calls occurred on August 24, 2016. Figure 4 shows the flow of patients in both phases of the study. Due to inconsistent recording of excluded patients, the number of patients that did not match each inclusion criterion is unknown. Recruitment ended in April 2016 due to completion of the allocated funding period. Measurement data from patients in the observation group and the pilot RCT are shown in Tables 2 and 3 respectively.

**Table 2:** Values on measures amongst participants in the observation phase at each time point

Outcome	Baseline		3 weeks		12 weeks/3 months	
	*M* (SD)	Median (range)	*M* (SD)	Median (range)	*M* (SD)	Median (range)
Performance-based						
Grip strength (Kg)						
Right	17 (12.4)		18 (13.7)			
Left	14 (11.4)		12 (12.3)			
10-m walk test (s)	41 (28.4)		52 (52.7)			
MoCA	14 (6.2)	13 (4–26)	17 (4.8)	18 (7–25)		
BI	11 (4.5)	10 (4–20)	11 (3.5)	11 (6–17)		
Self-report						
FSS		43 (15–63)		51 (11–61)		
VAF-S		6 (0–10)		7 (2–10)		
EQ-5D-5L VAS	57 (26.2)	60 (10–100)	59 (26.8)	65 (0–100)	56 (16.5)	60 (30–85)
RMI		6 (1–14)		8 (1–12)		9 (0–15)
WHODAS		45 (18–83)		53 (33–77)		44 (12–100)

Data were included only if the patient successfully completed assessment.

Abbreviations: *MoCA*, Montreal Cognitive Assessment (Mandarin version); *BI*, Barthel ADL Index; *FSS*, Fatigue Severity Scale; *VAF-S*, Visual Analog Fatigue Scale; *EQ-5D-5L*, EuroQol Health Questionnaire, including the EQ VAS (Visual Analogue Scale); *RMI*, Rivermead Mobility Index; *WHODAS*, World Health Organization Disability Assessment Schedule (12-item). *M* (*SD*), mean and standard deviation. WHODAS scores represent a percentage of the maximum disability score (the total score, between 0 and 48, is divided by 48 and multiplied by 100)

**Table 3:** Values on measures amongst participants in the pilot randomised controlled phase at each time point

Outcome	Baseline				3 weeks				12 weeks/3 months			
	Feedback		No Feedback		Feedback		No feedback		Feedback		No feedback	
	*M* (SD)	Median (range)	*M* (SD)	Median (range)	*M* (SD)	Median (range)	*M* (SD)	Median (range)	*M* (SD)	Median (range)	*M* (SD)	Median (range)
Performance-based												
Grip strength (Kg)												
Right	21 (13.2)		24 (13.1)		20 (13.7)		25 (13.6)					
Left	19 (11.7)		17 (11.2)		23 (9.3)		15 (10.1)					
10-m walk test (s)	43 (31.8)		40 (32.3)		37 (34.5)		48 (41.8)					
MoCA	17 (5.6)	18 (9–28)	17 (6.4)	21 (7–26)	19 (7.4)	22 (6–28)	18 (6.4)	21 (7–26)				
BI	9 (4.0)	8 (5–18)	11 (4.2)	11 (5–21)	12 (3.7)	12 (6–19)	12 (4.4)	13 (7–20)				
Self-report												
FSS		39 (9–50)		48 (9–60)		40 (33–55)		52 (20–63)				
VAF-S		6 (0–10)		8 (0–10)		5 (3–10)		7 (5–10)				
EQ-5D-5L VAS	47.9 (24.9)	52 (10–90)	55 (29.6)	60 (0–100)	52 (24.5)	57 (0–80)	61 (26.0)	70 (20–100)	55 (24.2)	50 (30–90)	60 (10.2)	60 (50–75)
RMI		4 (1–13)		6 (2–15)		8 (1–14)		6 (3–15)		8 (3–15)		9 (0–14)
WHODAS		48 (12–85)		54 (16–87)		46 (4–79)		44 (20–95)		39 (2–60)		45 (4–79)

Data were included only if the patient successfully completed assessment.

Abbreviations: *MoCA*, Montreal Cognitive Assessment (Mandarin version); *BI*, Barthel ADL Index; *FSS*, Fatigue Severity Scale; *VAF-S*, Visual Analog Fatigue Scale; *EQ-5D-5L*, EuroQol Health Questionnaire, including the EQ VAS (Visual Analogue Scale); *RMI*, Rivermead Mobility Index; *WHODAS*, World Health Organization Disability Assessment Schedule (12-item); *M*, mean; *SD*, standard deviation. WHODAS scores represent a percentage of the maximum disability score (the total score, between 0 and 48, is divided by 48 and multiplied by 100)

### Completeness of data

It is obvious that few patients across both phases had complete clinical assessment data across all measures even at discharge (see Table 4). Lost data were greater at 3 months follow-up. Eleven patients across both phases did not receive a second assessment and 3 months follow-up due to reduced communication between the ward and research office. One patient in the pilot RCT phase completed the intervention without completion of clinical assessments at any stage. Incomplete data at baseline (for example weight, height, 10MWT data and grip strength) can be partly explained by patients being unable to stand and weakness in their affected side. Thirteen patients (five in the observation phase and eight in the pilot RCT phase) were unable to walk. Seven patients in the observation phase were unable to complete the grip strength assessment due to weakness on their left side. Detailed reasons for incomplete baseline data for the FSS, VAF-S and WHODAS were not recorded. Discharge dates were not recorded for the majority of patients in the observation group (*n* = 16), although this was collected for all RCT patients.

**Table 4:** Completion rates of measures amongst participants in the observation and pilot randomised controlled trial phase

Completion	Observational phase (*n* = 20)		Pilot RCT Phase (*n* = 30)			
			Feedback (*n* = 14)		No feedback (*n* = 16)	
	Full (100%)	Completion range	Full (100%)	Completion range	Full (100%)	Completion range
Performance-based outcomes (*n* = 5)*						
Baseline	*n* = 3	*n* = 2 65–75%	*n* = 2	*n* = 3 57–78%	*n* = 0	*n* = 5 75–93%
3 weeks	*n* = 0	*n* = 5 55–70%	*n* = 0	*n* = 5 50–85%	*n* = 0	*n* = 5 62–75%
12 weeks						
Self-report outcomes (*n* = 5)^^^						
Baseline	*n* = 2	*n* = 3 90–95%	*n* = 2	*n* = 3 85–92%	*n* = 0	*n* = 5 87–93%
3 weeks	*n* = 0	*n* = 5 65–70%	*n* = 0	*n* = 5 78–85%	*n* = 0	*n* = 5 75%
12 weeks (*n* = 3)	*n* = 0	*n* = 3 35–45%	*n* = 0	*n* = 3 42–50%	*n* = 0	*n* = 3 50%
Smartwatch activity data						
Patient data recorded by watch and downloaded by ward staff	*n* = 20	100%	*n* = 14	100%	*n* = 16	100%
Smartwatch data recorded in log	*n* = 4	20%	*n* = 14	100%	*n* = 16	100%

Completion range refers to the percentage of recruited patients whom successfully completed assessment across measures

*Grip strength left, grip strength right, 10-m walk test; *MoCA*, Montreal Cognitive Assessment (Mandarin version); *BI*, Barthel ADL Index

^*FSS*, Fatigue Severity Scale; *VAF-S*, Visual Analog Fatigue Scale; *EQ-5D-5L*, EuroQol Health Questionnaire, including the EQ VAS (Visual Analogue Scale); *RMI*, Rivermead Mobility Index; *WHODAS*, World Health Organization Disability Assessment Schedule (12-item)

Despite the loss of patients, the data quality for the patients that were assessed at discharge and 3 months post-discharge follow-up was good. Complete data were collected on WHODAS, MOCA, Barthel ADL Index, RMI and EQ-5D-5L measures from all 39 patients assessed at discharge. One patient did not cooperate on the additional measures. Similar to baseline, seven patients could not complete the 10-m walk test at discharge due to an inability to walk. At 3 months post-discharge follow-up, the majority of patients that were contactable completed all three assessments (RMI, WHODAS and EQ-5D-5L measures), 21 out of 24 patients recorded.

The majority of patients in the observation phase (see Table 4) were not recorded in the smartwatch log leading to no record of distribution or functioning of each smartwatch. Duplicate and mislabelled data may be partly explained by poor understanding of the protocol for downloading smartwatch data in the initial phase of the study, for example, how to customise the watch with the patient ID. Smartwatch data were consistently recorded and collated after the study progressed into the pilot RCT phase. Completeness of smartwatch data also improved from the observation phase to RCT. Only eight weekdays of activity data were lost in the RCT compared to 83 weekdays in the observation phase.

No valid gait data were recorded due to initial problems with the functioning of the sensor (faulty Bluetooth connection) and inconsistent recordings of leg length and shoe size. Oxford Brookes visited the hospital to help rectify these problems which have now been amended to allow gait data to be recorded for any future large-scale RCT. Activity data were returned from all 50 patients (*n* = 20 observation phase and *n* = 30 RCT pilot phase).

During the observation phase, duplicate data files were common (see methods) with data files from certain days not correctly labelled with the patient identification number (ID); in response to this, the initial process for downloading and transferring smartwatch data was altered. Each patient’s smartwatch data were downloaded daily (as a precaution against loss of data in the watch), but data were not removed from the watch until the end of the intervention. Thus, the last download gave one complete smartwatch data file per patient covering the whole intervention (instead of a data file for each day of the intervention). This allowed for easier collection and understanding of the data when arriving in Oxford.

### Adherence rate to wearing the smartwatch

Patients often forgot to wear their smartwatch, but this problem was improved during the pilot phase by ward staff providing regular reminders. Adherence rates for wearing the watch (for all four periods) improved from 14% during the observation phase to that of 74% in the feedback and 86% in the no feedback group. Patient adherence to wearing the watch is summarised in Table 5.

**Table 5:** Patient adherence to wearing the smartwatch monitor

	Trial Days^a^	Periods worn for in days (percentage)^b^				
		4 (All periods)	3	2	1	≥ 1
Observation	450 (328)	49 (14.9%)	145 (44.2%)	34 (10%)	17 (5.2%)	245 (74.7%)
Pilot RCT-feedback	240 (175)	131 (74.4%)	38 (21.6%)	6 (3.4%)	1 (0.6%)	176 (100.6%)
Pilot RCT-no feedback	216 (152)	131 (86.2%)	23 (15.1%)	4 (2.6%)	1 (0.7%)	159 (104.6%)

^a^Total days (weekdays). ^b^days worn divided by weekdays; multiplied by 100 to get percentage

### Adherence to protocol

Ward staff actively took part in both phases of the study despite no financial reward. Overall, in both phases, recruitment adhered to the inclusion criteria, apart from age, whereby individuals under the age of 45 and over the age of 75 were recruited, and stroke onset, whereby five patients in the observation phase and one in the feedback group were recruited with a stroke onset of more than 4 months. In addition, grip strength in both the unaffected and affected arm was measured.

In addition to weekdays, activity was also recorded on weekends leading to an increase in data than originally expected. The total number of days of activity data recorded per group, including and excluding weekends, is summarised in Table 4. The range of intervention length was greatest in the observation group (5–45 days) but matched between the feedback (2–29 days) and the no feedback group (3–23). The length of stay in the observation group was estimated from the smartwatch data as minimal discharge dates were given.

During the pilot RCT phase, patients were randomised into the feedback or no feedback group effectively. However, during the 3-month observation visit (see methods), it was apparent that the functioning and feedback system of the smartwatch was not consistently explained to patients. It is therefore possible that patients within the feedback group will not have fully understood or been aware of the function of their smartwatch in relation to their activity.

### Readiness to randomise

All patients in the pilot RCT phase were correctly randomised into each group (see Fig. 4) and provided with the correct smartwatch (that provides feedback or does not provide feedback) as validated in the smartwatch log.

### Potential for effect

**Fig. 5:**
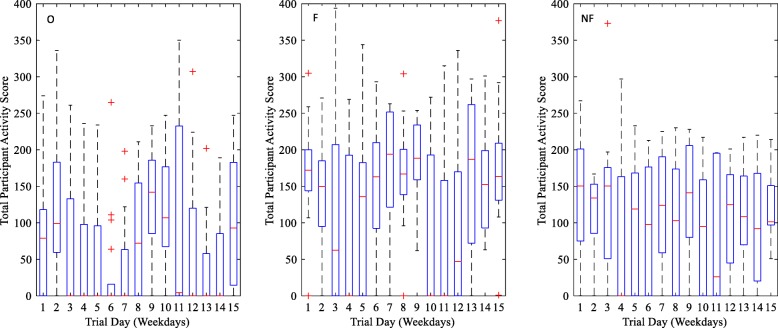
Box plots showing the variance of activity score amongst participants in the observation (O), feedback (F) and no feedback (NF) group across 15 weekdays. + Crosses indicate outliers

**Fig. 6:**
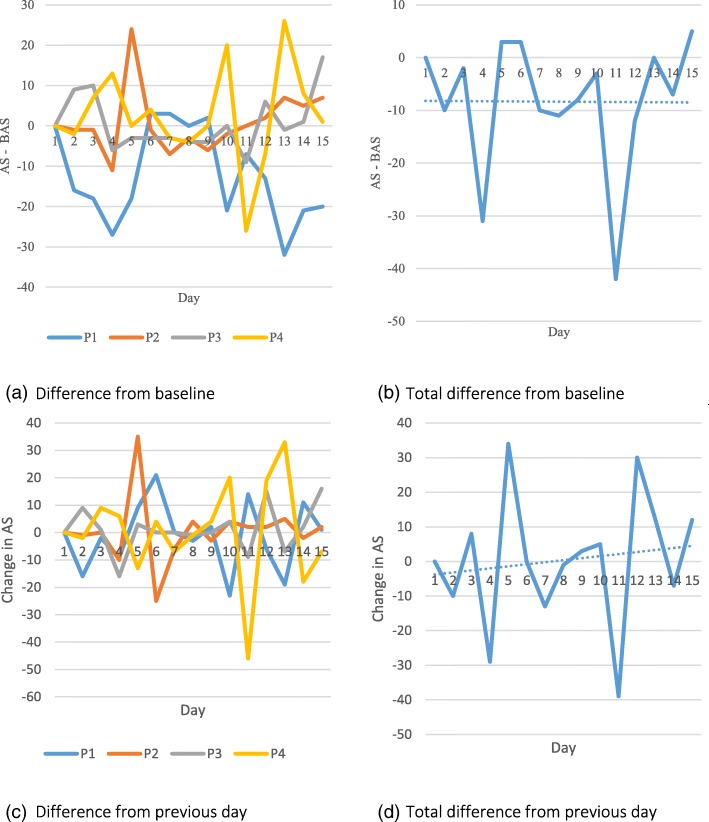
Graphs showing different analysis metrics of activity score (AS) from participant 1033 (Pilot RCT-No Feedback Group). Graphs (**a**) and (**b**) show the difference in AS from baseline AS (BAS). Graphs (**c**) and (**d**) show the difference in AS from the previous day’s values. P1 to P4 refer to each 2-h time period between 08:00 and 16:00

**Fig. 7:**
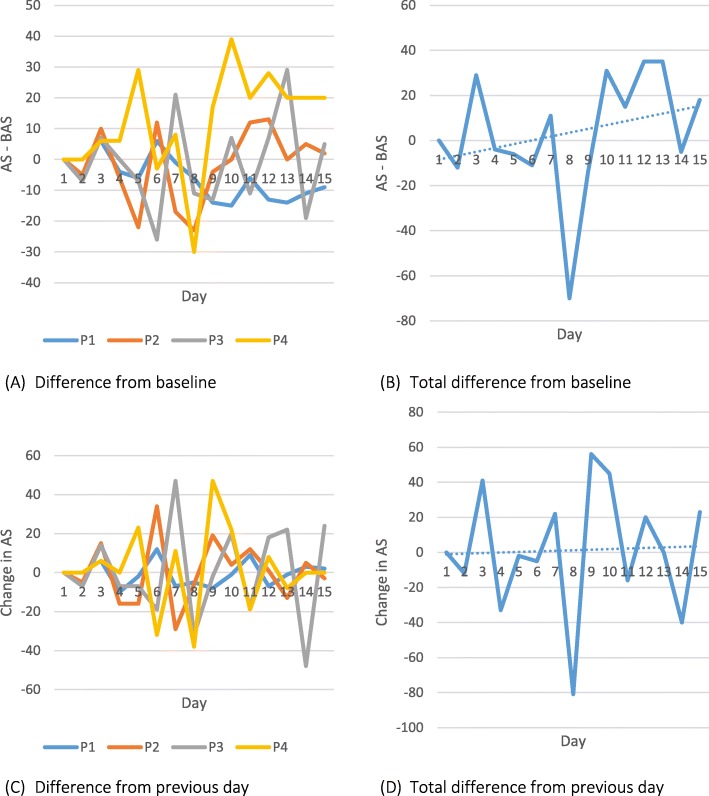
Graphs showing different analysis metrics of activity score (AS) from participant 1029 (Pilot RCT-Feedback Group). Graphs (**a**) and (**b**) show the difference in AS from baseline AS (BAS). Graphs (**c**) and (**d**) show the difference in AS from the previous day’s values. P1 to P4 refer to each 2-h time period between 08:00 and 16:00

Only activity recorded during time periods of 1 to 4 (representing the time period between 08:00–16:00) were included in analysis due to the limited battery life and thus variance in duration of time period 5. When comparing daily activity levels, the feedback group were more active on average; however, there was more variability in the control (no feedback) group at baseline (see Table 6). A summary of activity levels per group per time point is shown in Table 7 (and visually in Fig. 5). Analysis metrics of activity scores across the intervention for an individual patient within the control and feedback group are shown in Figs. 6 and 7 retrospectively.

**Table 6:** Activity recorded per group in comparison to baseline

	Collected data days^a^	Number of days that activity exceeded baseline	Rate of increased activity (%)^b^
Observation	328 (245)	207	63.1 (84.5)
Pilot RCT-feedback	175 (176)	115	65.7 (65.3)
Pilot RCT-no feedback	152 (159)	87	57.2 (54.7)

^a^Expected (actual). Expected day refers to the total number of weekdays from when a patient started the trial and when they left the trial. This is then summed over the group. Actual refers to the actual number of weekdays that data were collected

^b^Total number of days that activity levels exceeded baseline divided by total expected and actual (in brackets) days; multiplied by 100 to get percentage

**Table 7:** Activity score across the intervention per experimental phase (median < quartile 2–quartile 3 > [N])

	Observation	Pilot RCT	
		Feedback	No feedback
Baseline	79 < 0–118 > [20]	172 < 144–200 > [14]	150.5 < 75–201 > [16]
Day 5	0 < 0–96 > [17]	136 < 0–182.5 > [11]	119 < 0–168.25 > [15]
Day 10	107 < 67.5–176.75 > [17]	0 < 0–193 > [10]	95 < 0–159 > [12]
Day 15	93 < 14.5–182.75 > [15]	163.5 < 131–209 > [10]	101.5 < 97–151 > [6]

The results from the smartwatch activity data are clearly very preliminary and show that it is feasible to obtain the data, but due to the high inter-personal variability (as would be expected), these results do not show any differences and therefore any further statistical analysis would be misleading at this stage. Due to an incomplete and extremely variable dataset (see Fig. 5 and Additional file 2), no further statistical analyses were conducted as the data is underpowered.

The shortcomings and issues reported in the discussion which resulted in this dataset can be addressed; after which, a full study would be able to answer the clinical questions with a significant expansion in sample size.

No feedback was reported by the ward staff or research team despite prompts. One participant refused to wear the watch; however, no adverse effects or side effects of wearing the watch were reported.

## Discussion

This pilot study identified the challenges and support needed by ward staff and researchers and examined the feasibility of conducting an RCT using smartwatch activity monitors in research-naive rehabilitation wards. The main challenges when conducting this research were acquiring research data on a consistent sustainable basis, maintaining effective communication between the research and ward teams, ensuring clear understanding of roles and language barriers between the Chinese and UK teams. Frequent support was required in the form of email reminders to transfer assessment data, fortnightly teleconference calls and regular surveillance of incoming data. Delivery of a smartwatch RCT is feasible in a research-naive rehabilitation ward (in terms of recruitment rate, retention, adherence to wearing the watch, readiness to randomise and potential for effect); however, frequent support and guidance of research-naive staffs are required to ensure completeness of clinical assessment data and protocol adherence (specifically watch instructions). Recommendations for a follow-on RCT include an increase in the number of wards to aid recruitment, increasing the number of attempts to conduct 3 months post-discharge follow-up, weekly surveillance of incoming data to ensure data quality and the addition of a UK-based bilingual (English and Mandarin) research member to aid communication.

As in a number of trials, we found that although there are many patients admitted to the busy general hospital for stroke rehabilitation and despite the simple broad recruitment criteria, it proved difficult to recruit many patients. Retention rates were adequate at discharge despite the lost patients; however, telephone follow-up rates were low. Despite these difficulties, the ward staff did participate actively in recruitment and in delivery of the intervention undertaken as part of their daily work (they were not employed as or paid for being researchers), and over time, the quality of data improved. Patients exhibited high adherence to wearing the smartwatch, and activity data were collected and transferred effectively. On average, patients that received feedback did exceed their baseline activity level on more days than those that did not receive feedback, indicating a potential for effect.

Assessment data at baseline were well executed with minimal data loss, but completeness of data was much less at discharge or at the end of the intervention due to lost patients. The research staffs were capable of conducting the clinical assessments to a good standard; however, difficulties were shown in the recording and transferring of the data itself. The majority of missing data from the second assessment can be accounted for by patients being discharged from the ward before assessments could take place or without the research team being notified. Practice of procedures during the observation phase of the study alongside the simplicity of the protocol will have helped maintain data loss. The observation phase also allowed time to solve or highlight unforeseen problems prior to the RCT (e.g. the specific method of how to download smartwatch data and errors with the Bluetooth on the gait sensor). Despite this, a greater emphasis on the importance of collecting all required data and full comprehension of the patient on their role in the study could discourage such gaps in data. A better system of notifying research staff of planned and actual discharges should help. The presence of a qualified and experienced member of staff with responsibility for ensuring the collection of all assessments would better support data collection fidelity.

The outcome measures utilised could feasibly be implemented in the research setting in follow on studies as detailed below. Compliance to collecting the smartwatch data and wearing the watch was high, suggesting that it is a feasible method to record patient activity. There were missing data on outcome measures; however, this was mostly due to lost patients from poor communication rather than the ability to complete/conduct the assessments. Complete measures of cognition, independence in daily activities and health-related quality of life were collected for all of the 39 patients that were assessed at discharge. Some loss in arm function (grip strength) and mobility (10MWT) data were due to weakness or an inability to walk. Despite missing walk data, the other measure of mobility, Rivermead Mobility Index, had complete recorded data for all patients assessed at baseline, discharge and 3 months post-discharge follow-up, indicating that it is feasible to calculate mobility in a follow-on trial using this measure if the problem of lost patients is resolved. At 3 months post-discharge follow-up, the majority of patients that were contactable completed all three assessments (RMI, WHODAS and EQ-5D-5L measures). Therefore, the measures used seem to be appropriate for the aims of the study, and rather, the issue that needs to be addressed for a follow-on main trial is the likelihood of contact at the 3-month follow-up rather than suitability of measures.

The follow-up at 3 months had to be by (mobile) telephone, because patients do not routinely attend an out-patient clinic and to travel back specially would not be easy, especially as costs could not be reimbursed. This explains the lower rate of data collection. However, with better planning such as:

- Specifically mentioning the fact of a phone call at discharge, with a written note and verbally to patient and family
- Sending a reminder text 1 week beforehand, also offering a number to phone
- Allowing three attempts to make contact

it might be possible to acquire more data. Other means include online forms; post is not sufficiently reliable to be an option. To encourage larger follow-up rates, these changes are recommended for any future large-scale RCT.

To achieve minimal loss of data, a greater emphasis on the importance of feedback between the ward staff and the research assessors about patient progress through the trial, protocol deviation and the effectiveness of data collection procedures is recommended. For example, uploading the assessment and smartwatch data collected in China onto a secure, interactive online web space that could be assessed daily by the UK team would allow the data to be checked as it is updated in real-time. Previous multi-centre research has used interactive platforms allowing access to up-to-date data, as well as information regarding design, consent and verification of trial events to reduce the time taken to identify and resolve data-related queries and errors [36].

The lack of collected data regarding recruitment numbers and specifically the numbers of patients that did not match the inclusion criteria is a major limitation of this study. Patients in the study were confirmed to have met the eligibility criteria as recorded in the data collection forms; however, no information was recorded on the number of patients that were ineligible and the reasons why. The current study has therefore not informed a follow-on main trial on the reason behind the low-recruitment numbers, and thus the potential of recruitment bias cannot be assessed. An additional limitation of the study was that any occurrences of research assessors becoming unblinded to the patients group (be it feedback or no feedback) were not recorded, and therefore it is unknown whether this unblinding could have biased the clinical assessments. For a follow-on trial, the importance of the eligibility/screening log should be reinforced to all staff involved in the study and also during each fortnightly teleconference call.

Effective communication between the Chinese research team and the Chinese ward teams on the one hand and the English research teams (Oxford Brookes and Warwick) on the other hand were one of the main challenges within this study. As an international study, the challenges included language barriers, time differences which affected scheduling of Skype meetings and practical difficulties as direct face-to-face research input and explanation was not possible at short notice or on a regular basis. Nonetheless, regular Skype meetings were effective in raising concerns and queries, although time consuming due to the need for frequent translations. For a follow-on trial, a bilingual (English and Mandarin) research member is advised to aid comprehension during the meetings and during data transfer.

The ward staff initially did not adhere to the protocol concerning the smartwatch. During an observation visit by the UK teams, ward staff provided smartwatches to each group but they did not consistently explain the function and purpose of the watch. One reason may be that daily schedules of ward staff and time restraints meant that many staff were involved, often not really knowing what to do. The communication of complex research protocols across languages may have led to lack of clarity in the implementation of procedures. Despite this, smartwatch data were collected to a high standard with data collected from all patients.

One consideration when evaluating this intervention is the possible impact of the Hawthorne effect (also referred to as observer effects); the alteration of a participant’s behaviour caused by their awareness of being observed (for a review see [37]). All patients are likely to have exhibited increased levels of activity than if they were not provided with smartwatches monitoring their activity, regardless of whether feedback was provided. The aim of the smartwatch is to evoke a change in behaviour, specifically an increase in movement, due to the monitoring and provision of activity feedback. Therefore, any observer effects are expected and can be seen as a result of the research design rather than a bias that would hinder the effectiveness of the intervention. As monitoring and thus possible observation effects occurred in both experimental groups, the impact of feedback specifically can still be determined by comparing the feedback and no feedback groups. However, it appeared that the ward staff did not appear to consistently explain the monitoring system of the smartwatch to patients which may have led to a different understanding of the purpose of the smartwatch and thus different observer effects between patients and/or groups.

The lack of an independent measure to monitor whether the watch was worn was a major limitation of the model of smartwatch used. The smartwatch produces only an arbitrary measure of whether the watch was being worn; using an algorithm based on movement and probably underestimates the time worn, for example, if the patient was lying quietly in bed. The addition of a temperature sensor or pulse rate monitor could overcome this, increasing the validity of the activity measure. In addition, the smartwatch only produces processed data, due to its limited storage capacity. A larger storage capacity would allow direct access to raw activity data after its collection, and thus open wider opportunities of data analysis, for example, producing movement graphs [31].

The time-frame with which the activity is recorded (08:00–17:00) may also bias the activity levels recorded. The local ward routine alongside medical and rehabilitation schedules limits the time available for patients to alter their activity levels. Indeed, it is precisely the evenings and weekends that offer the biggest opportunities for increased patient activity. A smartwatch with a battery life able to record from morning to night and over 2 or 3 days would provide a richer account of activity changes and much more likelihood of targeted feedback altering behaviour. One possibility to increase the amount of activity recorded is for patients to wear and charge their own smartwatch over the weekend. Future research would need to investigate whether charging of the smartwatch by patients is feasible, for example, in terms of data loss, protocol compliance and maintaining randomisation of groups.

Whilst the authors recognise that the smartwatch does have some shortcomings, it must be noted that it is the best device for meeting the requirements of the project, when compared to commercial activity monitors or paired-to-smartphone smartwatches. That is, that it provides the sensing capacity, on-board processing, and user interaction capabilities to monitor and provide real-time feedback as a single standalone device. These capabilities are not otherwise available, and if the limitations were overcome, the smartwatch performance would be greatly enhanced. The use of accelerometers as a measurement of activity within stroke patients is a widely used method, generating valid and reliable insights into activity levels [38–40]. Nevertheless, it has yet to be shown that feedback from accelerometers leads to actual long-term change in behaviour amongst this population.

Despite the reported difficulties in data collection and recruitment, conducting clinical trials within developing/research-naive settings, such as many hospitals in China, is becoming more prevalent. In comparison to Europe and North America, research in developing countries offer smaller running costs and large numbers of patients, often due to higher rates of disease as well as larger populations, allowing for large scale clinical trials [41]. Conducting the present trial within Anhui hospital in China did indeed provide a large pool of stroke patients from which to recruit, but final recruitment numbers were in comparison small. An increase in the number of recruitment wards (eight wards in total) is recommended for a follow-on RCT.

In order to emphasise and maintain the importance of strong adherence to protocol, future studies conducting similar research within a research-naive setting are advised to provide frequent support and training by experienced and qualified researchers alongside separate experimental phases to determine the feasibility of assessment and randomisation. An observation phase prior to randomisation allows a period to implement measures, increasing understanding and sound practice of protocol procedures to better prepare and maintain a high-quality randomised intervention. Additional considerations from this research include the selection of a smartwatch that meets the functional requirements of the project whilst possessing a maximum battery life that complements the duration of data collected, and data that can be checked and assessed as it is being collected by both research teams in order to maintain data quality. Adjustments required for a follow-on RCT include increasing the number of wards to aid recruitment, increasing the number of attempts to conduct the 3 months post-discharge follow-up, weekly surveillance of incoming data to ensure data quality and the addition of a UK-based bilingual (English and Mandarin) research member to aid communication.

## Conclusion

Activity levels of stroke survivors undergoing rehabilitation are commonly below the recommended levels for optimal recovery. Tailored feedback of physical activity is a possible method to encourage greater levels of activity and thus positive clinical outcomes post-stroke. The aim of this feasibility study was to identify the challenges and support needed by ward staff and researchers and to examine the feasibility of conducting an RCT using smartwatch activity monitors in research-naive rehabilitation wards. Delivery of a smartwatch RCT is feasible in a research-naive rehabilitation ward; however, frequent support and guidance of research-naive staff is required to ensure completeness of clinical assessment data and protocol adherence. Adjustments required for a follow-up RCT include increasing the number of wards to aid recruitment, increasing the number of attempts to conduct 3 months post-discharge follow-up, weekly surveillance of incoming data to ensure data quality and the addition of a UK-based bilingual (English and Mandarin) research member to aid communication.

## Additional files

Additional file 1: ZGPAX S8 Android Smartwatch Specifications. (DOCX 12 kb)

Additional file 2: Distribution plots showing distribution of total daily activity scores for Feedback group (A), No Feedback (control) group (B), observation group (C) and all groups (D). Table 7 shows a summary of this data at days 1, 5, 10 and 15. Red crosses indicate the mean and green squares the median. (DOCX 96 kb)

## Abbreviations

RCT: Randomised controlled trial
SWAFT: Smart Watch Activity Feedback Trial Committee
