# Supplementary material for: Evaluation of a smartwatch-based intervention providing feedback of daily activity within a research-naive stroke ward: a pilot randomised controlled trial
Source: Pilot Feasibility Stud. 2018 Oct 6;4:157. doi: 10.1186/s40814-018-0345-x (PMC6173888; doi:10.1186/s40814-018-0345-x)
Supplement: Supplementary file 1 — ZGPAX S8 Android Smartwatch Specifications. (DOCX 12 kb) [file 40814_2018_345_MOESM1_ESM.docx]

**Additional file 1.** ZGPAX S8 Android Smartwatch Specifications.

| Hardware Specifications for the ZGPAX S8 Android Smartwatch | |
| --- | --- |
| Size | 58 × 42.5 × 13 mm |
| Weight | 67 g |
| Screen | 40 mm capacitive touchscreen |
| Battery | 3.7V/470mAh Li-ion (rechargeable) |
| CPU | MTK6572 Dual Core 1.2 GHz |
| Memory | 12 GB total |
| RAM | 512 MB |
| Android Version | 4.4.2 |
| Sensors | GPS, Accelerometer |
| Connectivity | WiFi, Bluetooth, 2G, 3G |
| Waterproof | No |

Information retrieved from: http://zgpax.com/androidwatch/41-36.html
