# Supplementary material for: Evaluation of a smartwatch-based intervention providing feedback of daily activity within a research-naive stroke ward: a pilot randomised controlled trial
Source: Pilot Feasibility Stud. 2018 Oct 6;4:157. doi: 10.1186/s40814-018-0345-x (PMC6173888; doi:10.1186/s40814-018-0345-x)
Supplement: Supplementary file 2 — Distribution plots showing distribution of total daily activity scores for Feedback group (A), No Feedback (control) group (B), observation group (C) and all groups (D). Table 7 shows a summary of this data at days 1, 5, 10 and 15. Red crosses indicate the mean and green squares the median. (DOCX 96 kb) [file 40814_2018_345_MOESM2_ESM.docx]

(A) Feedback Group

(B) No feedback (control) Group

(C) Observation Group

(D) All Groups

**Additional file 2.** Distribution plots showing distribution of total daily activity scores for Feedback group (A), No Feedback (control) group (B), observation group (C) and all groups (D). Table 7 shows a summary of this data at days 1, 5, 10 and 15. Red crosses indicate the mean and green squares the median.
